# Quality of Life: Information and Learning Resources in Supporting People with Severe Life-Changing Injuries to Return to Independence

**DOI:** 10.1100/tsw.2004.110

**Published:** 2004-07-20

**Authors:** Bernadette Cassidy, Alan Clarke, Said Shahtahmasebi

**Affiliations:** ^1^ Library and Learning Coordinator, Christchurch Polytechnic Institute of Technology (CPIT), P.O. Box 540, Christchurch, New Zealand; ^2^ Allan Bean Centre, Christchurch, New Zealand; ^3^ School of Mathematics and Statistics, Faculty of Health and Sciences, Christchurch Polytechnic Institute of Technology (CPIT), P.O. Box 540, Christchurch, New Zealand

**Keywords:** depression, quality of life

## Abstract

This paper reports the first stages of the development of an integrated care model for people with massive sudden change in their lives with special reference to spinal cord injuries. The model aims to be holistic by placing the patient at the centre of the service. In addition to providing medical care for physical injuries, the model emphasises at the outset that regaining a good quality of life postinjury is expected. There is quality of life after change if individuals and their support network are provided with access to quality information and evidence for them to make informed choices. In New Zealand, this has been referred to as “life beyond bugger”!

## INTRODUCTION

A serious injury (such as spinal cord injury) creates a massive and sometimes sudden change in a person's life. Over and above the physical injuries and loss of functions, the resulting mental and emotional trauma can lead to significant impairments, e.g., post-traumatic stress and emotional distress[1], diminished life satisfaction, low morale and self-esteem, depression[2,3,4], and suicidal ideation[5] resulting in a perceived lowered overall quality of life. A feedback effect may be assumed. A lowered overall quality of life will have a significant effect on the patient's recovery and rehabilitation.

The process of physical healing on its own may be an acceptable medical care model in general. Such a model assumes that full recovery will follow survival. In a series of papers, Ventegodt and colleagues[6,7,8,9,10,11] suggest that the perceived quality of life is a complex process and depends on many social, health, and dependency measures and individual characteristics. Therefore, physical healing alone (surviving the injury), with the loss of psychological and physical independence, will delay the process of a full recovery.

Although the direction of causality between health and quality of life is vague in the general population, in the patient population it is reasonable to assume a lowered quality of life as a consequence of ill health or injury, which in turn will have an effect on the recovery from ill health. For example, Ventegodt and colleagues[12] report that health outcomes can be improved by adding quality to the lives of patients with chronic illnesses. The question is what, how, when, by whom, and what resources are required to care for the “whole” patient? The conventional medical model of attending to the physical injuries of patients delegates the job of rebuilding and fitting back into society at a later stage to the patients and their families who are already traumatised by the change in their lives. Specifically, in cases where massive change has occurred, a more comprehensive approach is required to give the patient control over their own rehabilitation.

We are reminded of an analogy of the case of a patient being treated for complex mental and physical symptoms[13]. The patient had exhibited a range of psychological symptoms: he insisted on shifting the mountains on the outskirts of the city with his bare hands. His family had consulted well-known consultants and specialists and all manner of tests had been conducted and treatments had been prescribed, but to no avail. The patient's condition deteriorated; he became emaciated and skeletal, and as a last resort his family had tied him to the bed for his own safety. In desperation, the family consulted a doctor rumoured to have healing powers; they were hoping for a miracle. After talking with the family and checking the patient’s medical history, the doctor asked for the patient to be untied. The doctor told the patient that he had come to ask for his help to shift the mountains and had heard that he (the patient) is the only one that could do this. To the horror of the family, he helped the patient to get ready and they started to walk towards the front door. As the doctor reached for the door handle, he turned to the patient and said, “Let’s shake on our new partnership!” The doctor squeezed the patient’s hand so hard that the patient complained of pain. The doctor persuaded the patient that since his hands are important for this mission, they both should have something to eat and then try again the next day. The patient voluntarily went back to his room and waited to be fed. In the meantime, the doctor made up a comprehensive prescription (mainly dietary) and gave it to the patient's family. The same pattern of events followed the next day and the next and the next and so on. On what became the final visit, the doctor arrived at the house and examined the patient and said that he was now strong enough to go and help shift the mountains, to which the patient responded, “Why, are you crazy doctor!” In the final analysis, the patient’s perception of being in the driving seat may well have assisted with the final outcome.

In an unpublished paper[14], presented at the First National Conference of the Spinal Network “Life Beyond Bugger!” Clarke stressed that successful rehabilitation is hard work, involves personal learning, and is the responsibility of the recovering person. As the major stakeholder in the outcome, the recovering person is the “chairman of directors”. Everybody else is “staff”. Rehabilitation professionals, programmes, and centres need to recognise this and provide the resources and support to allow it to happen.

If the recovering person is to take responsibility to drive his/her own rehabilitation towards independence, then the provision of access to the “vehicle” must be complemented with a quality and well-defined map. Clarke[14] outlines the development of an integrated care model that aims to be holistic by placing the emphasis on the patient as a whole being, giving him or her control and real hope. In this paper, we report the development of the library and learning resources/information services “the quality map” to assist the “chairman” to make well-informed and evidence-based decisions.

## THE ALLAN BEAN CENTRE

Burwood Hospital in Christchurch is New Zealand's largest and most comprehensive rehabilitation campus and caters to spinal cord injuries, brain injuries, stroke, orthopaedic conditions, heart and chest problems, pain management, etc. Every year, more than 2,500 people recover from injury or illness at

Burwood. Rehabilitation starts from day one. Central to this approach is the Allan Bean Centre (ABC) for Research and Learning in Rehabilitation. For example, as soon as patients arrive in the Spinal Unit and their injuries are stabilised, they embark on a rigorous programme of physiotherapy and occupational therapy treatment as necessary. The rehabilitation process is essentially educational, not medical, and follows the *Independent Living Paradigm of Rehabilitation.* First proposed by DeJong[15], this is a model where the process of recovery is controlled by the "consumer", not by the professionals. At the ABC, this is referred to as the "New Rehabilitation", i.e., that the recovering person must take responsibility for his/her rehabilitation and recovery. The philosophy of the centre, "It's great to be alive", provides a pathway to a society that values ability and diversity and provides hope. "Health professionals are on tap, not on top!"

Some might ask whether this approach improves the chances for the very severely impaired person to achieve complete recovery. The standard (society's) view is that for a paraplegic or a tetraplegic, complete recovery is walking normally, having normal bladder and bowel functions, normal sex, no pain, and back to work in the same job, or something similar. This seldom happens. Much more often, the person "walks" with a wheelchair till death do part, needs expert care of bladder and bowel, finds more inventive ways of enjoying sex and having children, manages pain successfully, finishes up with a much better job than he/she had before, and is independent and happy. If the person also learns to manage society's view of disability, gets a wee bit cross at times, but is never angry, recovery is deemed to be completely successful. This goal will be achieved if the persons expectations are managed from day one with accurate information, "information intensive care", delivered to the person and to the frightened family with sensitivity. It really is Bernard Shaw's definition of education: knowledge wielded by love. Does early intervention with programmed "real" information interfere with the healing process? Not at all! It is a part of the healing process for which health professionals are only partly trained and are too busy with medical duties to deliver in any case. Lying in hospital is desperately boring and a successful system of delivering reality and hope during the endless hours of unoccupied time is essential. A fully equipped contemporary library and information resource, staffed by skilled and caring people, is as important in a rehabilitation facility as operating theatres, radiology departments, and laboratories are in hospitals. Without a contemporary information resource, rehabilitation is not holistic.

To achieve and establish this model for recovery, an old hospital campus had to be equipped with a "modern brain". This was achieved by the New Zealand Spinal Trust raising NZ$2 million for the ABC and the ABC is operated as a joint venture between the Burwood Hospital and the New Zealand Spinal Trust.

The ABC opened in October 2001. For the ABC to succeed, it needed to translate its philosophy into practice by adopting a holistic approach to address the spiritual and psychological needs of the patients. That is to treat the process of rehabilitation as a process of decision making by all involved, e.g., patients, practitioners, and support networks. This in turn necessitates access to quality information and evidence to inform the process of decision making. Therefore, a library and information resource centre is essential. Indeed, this was identified as such and referred to as the "powerhouse" of the ABC.

## The ABC Library and the Burwood Academy of Independent Living

The *Burwood Academy of Independent Living* has been established to be the vehicle for all research and learning activities on the Burwood campus. An executive committee of stakeholders (including the library and learning coordinator) aims to oversee all research and output from the academy, assist with research proposals, and organise seminars and conferences. Most people in a situation where they have had a serious illness or injury can very quickly enter a cycle of dependency. The aim with the *Burwood Academy of Independent Living* is to ensure a focus on independence and that the individuals will regain their lives, return to work, retrain, start a new hobby, and so on. The ABC Library is being developed in close collaboration with Christchurch Polytechnic Institute of Technology (CPIT), which has a memorandum of understanding with the Trust and the ABC to be the primary educational provider to recovering people at Burwood. Unlike medical libraries, the ABC Library is open to all: patients, families, whanau (informal support network), caregivers, as well as staff, students, researchers and the public and provides a range of resources and services.

## Services Developed in the ABC Library

For the library to be the “powerhouse”, the librarian had to meet the challenge of translating the philosophy of “the patient as the chairman of directors” into practice. The recovering person needs access to a range of resources whether it is information about their condition, pain management, or retraining and redirection. The model adopted by the librarian follows a dynamic approach of assessing the needs of its clients by continually consulting all stakeholders. The ABC is not a conventional rehabilitation model and, therefore, the library and information system has to meet the challenges of providing unconventional (as well as conventional), but appropriate and relevant, services. In addition to developing and implementing a range of learning and information services, the librarian coordinates access to ABC and other external resources/services. Specifically:

- Information: e.g., information on their condition, pain management, others with similar condition, how to manage their condition, and so on
- Newsletters: production of a regular digest based on a relevant single issue with contributions from staff and patients (being developed)
- ICT: e.g., use of the Internet for access to information, ICT training skills
- Assistive Technology aids: e.g., trackballs, voice recognition, Orbitouch (keyless keyboard)
- Vocational rehabilitation: e.g., career counselling, retraining, and support and course programmes
- Computers for Special Needs: e.g., computer training courses
- Involvement: a volunteer scheme, “Useful People” to engage people with various backgrounds in the development and provision of the library services, e.g., past and present patients to assist in the library
- Partnership: The development of the centre is a joint venture between the Canterbury District Health Board (CDHB) and the New Zealand Spinal Trust. CPIT is the primary educational provider and a partner in the development of the library. CPIT and the ABC have a memorandum of understanding to support and develop research and learning in rehabilitation. The library also has a good working relationship with the Canterbury Medical Library.
- Collections: The development of the collections is an on-going process. The ABC Library’s acquisitions reflect the needs of the research and rehabilitation community and include books, journals, videos, and CD-ROMs, as well as electronic resources. The ABC Library has established access to a number of collections including the Burwood Medical Library Collection, ABC Library Collection, Christchurch City Libraries Collection for patients, Burwood Hospital Fiction Collection, as well as the CPIT library.
- Communication: It is important that all stakeholders talk to each other and are clear about the philosophy and aims. The other key factor is establishing links with other organisations and community groups, e.g., Disability Information Service, Kiwiable, Disabled Persons Association, Parafed, the Champion Centre, etc.

Currently the library is staffed by a library manager/coordinator and a part-time library assistant whose salary is funded by the MacKenzie Trust. The ABC Library relies on donations from charitable trusts and other nongovernmental services for its resources and collections.

## ABC Library Users

The ABC Library has a diverse range of users, from patients, their families, whanau, and caregivers to staff, students, and researchers at Burwood and beyond. The users fall into two distinctive groups: patients and staff.

One aspect of the patients as users is to make them and their families comfortable with the idea of using the library services (though optional) as an integrated part of their recovery programme. Moreover, visiting the ABC Library could provide an informative alternative and escape from the rather restricting and clinical routines of physiotherapy and treatment regimes. When patients visit for the first time, they are not sure what to expect when they realize that they have the opportunity to not just browse the book collections, read the newspaper, watch a video, explore the Internet, write e-mails to their families, friends, and relatives, but that it can also be a gateway to accessing databases, courses, and services via CPIT. Early feedback comments from patients' sessions include: "Could the library be available at the weekends and in the evenings (it's so boring in the hospital/wards)?" "I want to learn how to use a computer." "Can we loan books from the public library?" As mentioned above, the ABC Library now has access to a monthly public library collection. In addition, the library is also available one evening per week with plans to extend the service to include weekends.

To maintain the flexibility and holistic nature of the service, it is important to understand the needs of the professional users too. Meetings were held with key staff members at Burwood, i.e., the Director of Nursing, Clinical Charge Nurses, Nurse Educators, Human Resources, Maori Health Coordinator, Physiotherapists, Occupational Therapists, etc. as well as key staff members at CPIT. It was important to get the staff on side, particularly the nursing staff. There was some wariness amongst the staff, some nursing staff have not quite grasped the idea that the ABC is primarily focused on the needs of patients. The usage by the nursing staff in particular has been slow, but is gradually growing, comments such as "The ABC is too far away, if only it was next door to the Brain Injury Rehabilitation Service." "We're too busy and haven't time to go there." "I thought it was only for the spinal cord injured." provided interesting insights. Clearly, communication has been an important area for development and has been integral to the service development from the outset.

Fortunately, the architects have done a wonderful job producing a building which takes one's breath away and which won a national award. Said one patient, "It is a lovely place to be in." Several medical staff have commented that it meets all their information and research needs.

## DISCUSSION

In New Zealand, all accidents are covered by the ACC (Accident Compensation Corporation) so motivation to recover is not greatly influenced by time taken for an insurance outcome. Due to the service being in its early developmental stages, as yet there are no measures of change or improvement in the quality of life of the patients. Measuring the quality of life is not an easy task; quality of life is a multidimensional process and requires a multimethod approach such as proposed by Ventegodt and colleagues[6,7,8,9,10,11]. One of the main problems in measuring change in the quality of life is that we have no knowledge of an individuals' perceived quality of life before the event. The first aim of the model is to minimise the depressing effect of the massive sudden change and help the patient acknowledge that there is life beyond. Specifically:

- Hope: An information pack issued to all patients and their informal support network (e.g., family members). The information pack emphasises and prepares the patients, with examples, that a normal life is achievable.
- Informed decision making: Provide relevant information for both the patients and their support network, and pointers how to find out more about their condition, list of services, others like them, etc. and how to formulate what they would like to do — there may be a need for retraining, or restructuring.

- Support services: Once patients have made a decision, they would invariably require support to achieve their goals. These services may be in the form of simply providing access to the Internet, (re-) training programmes, or putting them in touch with relevant formal support services (e.g., social services).

Hope, self-set goals, and support to assist individuals towards achieving their goals may often be the motivation to "complete" recovery despite massive lesions. A complete recovery may be considered through self-confidence and high self-esteem to lead an independent life, to learn new skills, to renew old skills, and to achieve life-long goals. The "mouth and foot" painters society can be viewed as a good example. There are examples of individuals who may have achieved "complete" recovery despite their conditions who now lead as normal a life as they could otherwise[2], e.g., developing a career, working "normal" hours, and sometimes achieving tasks that they would have not even considered in their previous life. The ABC strives to assist individuals to achieve their identified goals towards a self-paced independent living. The premise of the model has been to place the client in the driving seat. We do not claim to have achieved this, however. We would like to suggest that we have introduced positive changes in the lives of the clients in the right direction. For example, a cerebral palsy outpatient who only used to attend physiotherapy and the hydro pool is now a regular user of the library, despite setting him up with home access to the Internet and e-mail. Another example is the case of a stroke victim who led an active life prior to the stroke and now assists in the library as a volunteer.

However, there is some information in the form of service uptake statistics and comments to help design an integrated follow-up study of the patients going through the service. Whilst the number of users per month fluctuates slightly, the overall usage has an upward trend. Recent usage monitoring statistics show increased patronage of the ABC Library from 155 users in January 2003 to 586 users in March 2004. A brief breakdown of the data by main categories is shown in Table 1.

**TABLE 1**
**Aggregated Service Uptake**

| | Hospital Staff | Patients | Relatives | Visitors | Total |
|---|---|---|---|---|---|
| Jan-03 | 93 (60%) | 23 (15%) | 21 (13%) | 15 (10%) | 155 |
| Sep-03 | 186 (37%) | 102 (20%) | 60 (12%) | 16 (3%) | 503 |
| Mar-04 | 170 (29%) | 137 (24% | 114 (19%) | 23 (4%) | 586 |

The remaining categories include students and ABC staff. The database can provide higher levels of detail for each category, for example, hospital staff can be broken down by specialty: doctors, nurses, physiotherapists, occupational therapists, and so on. Since the recordkeeping began in January 2003, the number of patients/relatives has increased nearly six fold. Proportionally, over 40% of the uptakes are by patients and their relatives or their support network, which can be interpreted as a positive outcome, i.e., the model may be succeeding in getting the message across.

A lot has been achieved in the short term. The ABC Library has created interest from outpatients and community groups, and further work is necessary to expand on this. Funding is still a major issue to maintain the current momentum. Rehabilitation is of relatively low priority within the health service in New Zealand — the more is the pity. New models must prove themselves particularly if they are consumer driven: rehabilitation cannot be done to you. It is not therapy, it is "Do-It-Yourself (DIY)"

learning where the outcome is independence and happiness. Additional funding will be essential as the number of users increases and the demand for support services becomes more varied and demanding, not to mention the need for research funding within the new independent living paradigm.

Information about the different projects and activities currently in the Allan Bean Centre: the ABC Library, Patient Education, Useful People, Assistive Technology, Vocational Rehabilitation, Courses and Training, and Research can be found on the ABC website at www.burwood.org.nz

## References

1. Nielsen, M. (2003) Post-traumatic stress disorder and emotional distress in persons with spinal cord lesion. *Spinal Cord* **41**, 296–302.
2. Scherer, M.J. (2000) *Living in the State of Stuck: How Assistive Technology Impacts the Lives of People with Disabilities*. 3rd ed. Brookline Books, Cambridge, MA.
3. Cushman, L.A. and Scherer, M.J. (1998) Perceived Needs and Person-Technology Fit in Spinal Cord Injury. 12th Annual Conference of the American Association of Spinal Cord Injury Psychologists and Social Workers.
4. Krause, J.S., Crewe, N., and Kemp, B. (1999) Depression Among Individuals Living in the Community with Spinal Cord Injury: Incidence, Correlates, Case Studies, and Treatment. 13th Annual Conference of the American Association of Spinal Cord Injury Psychologists and Social Workers.
5. Kishi, Y., Robinson, R.G., and Kosier, J. (2001) Suicidal ideation among patients with acute life-threatening physical illness. *Psychosomatics* **42(5)**, 382–390.
6. Ventegodt, S., Merrick, J., and Andersen, N.J. (2003) Quality of life theory I. The IQOL theory: an integrative theory of the global quality of life concept. *TheScientificWorldJOURNAL* **3**, 1030–1040.
7. Ventegodt, S., Hilden, J., and Merrick, J. (2003) Measurement of quality of life I. A methodological framework. *TheScientificWorldJOURNAL* **3**, 950–961.
8. Ventegodt, S., Andersen, N.J., and Merrick, J. (2003) Quality of life philosophy VI. The concepts. *TheScientificWorldJOURNAL* **3**, 1230–1240.
9. Ventegodt, S., Andersen, N.J., and Merrick, J. (2003) Quality of life philosophy I. Quality of life, happiness, and meaning of life. *TheScientificWorldJOURNAL* **3**, 1164–1175.
10. Ventegodt, S., and Merrick, J. (2003) Lifestyle, quality of life, and health. *TheScientificWorldJOURNAL* **3**, 811–825.
11. Ventegodt, S., and Merrick, J. (2003) Measurement of quality of life VII. Statistical covariation and global quality of life data: the method of weight-modified linear regression. *TheScientificWorldJOURNAL* **3**, 1020–1029.
12. Ventegodt, S., Merrick, J., and Andersen, N.J. (2003) Quality of life as medicine: a pilot study of patients with chronic illness and pain. *TheScientificWorldJOURNAL* **3**, 520–532.
13. Shahtahmasebi, M. (1985) Treatment for a diagnosis or diagnosing a treatment. Personal communication.
14. Clarke, A. (2000) The Allan Bean Centre for Research and Learning in Rehabilitation at Burwood Hospital. The First National Conference of the Spinal Network "Life beyond Bugger!", Christchurch, New Zealand.
15. DeJong, G. (1979) Independent living: from social movement to analytic paradigm. *Arch. Phys. Med. Rehabil.* **60(10)**, 435–446.

## BIOSKETCHES

**Bernadette Cassidy** is the Library and Learning Coordinator at the Allan Bean Centre. She moved to New Zealand from the U.K. 2 years ago. She has worked as a professional librarian for 14 years in a number of information specialist posts. She is currently writing up her PhD research into the "Effects of

Information and Communication Technologies (ICT) on the Literacy Levels of Young People with Learning Difficulties and/or Disabilities”.

**Alan Clarke** is emeritus Professor of Surgery, Otago University, and currently Rehabilitation Advisor to the General Manager at Burwood Hospital, Christchurch, New Zealand. He was Dean of the Christchurch School of Medicine, Otago University, until 1994. As a result of a fall in 1991, he became a paraplegic. From 1994–2000, he was Director of the Spinal Injuries Unit at Burwood Hospital and was Executive Director of New Zealand Spinal Trust from 1995–2003. He has served on a number of national and international committees. His research interests are in the holistic healthcare provision. He loves the great outdoors and paints.

**Said Shahtahmasebi** is currently research methodology consultant and statistician at Christchurch Polytechnic Institute of Technology, Christchurch, New Zealand. His work experiences are mainly in the U.K. and cover a number of fields including public health, mental health, nursing, operational research, food, nutrition, and gerontology. His area of interests and expertise are related to the longitudinal modelling of health-related behaviour. He has set up the Good Life Project (http://lists.cpit.ac.nz/thegoodlife/) in New Zealand, which attempts to investigate food holistically.
